# Direct electrospinning of poly(vinyl butyral) onto human dermal fibroblasts using a portable device

**DOI:** 10.1007/s10529-018-2522-7

**Published:** 2018-02-15

**Authors:** Chih-Yao Chui, Pierre-Alexis Mouthuy, Hua Ye

**Affiliations:** 1 Institute of Biomedical Engineering, Department of Engineering Science, University of Oxford, Old Road Campus, Oxford, OX3 7DQ UK; 2 Botnar Research Centre, Nuffield Department of Orthopaedics, Rheumatology and Musculoskeletal Sciences, University of Oxford, Windmill Road, Oxford, OX3 7LD UK

**Keywords:** Electrospinning, Human dermal fibroblasts, Poly(vinyl butyral), Portable electrospinning device, Wound healing

## Abstract

**Objective:**

To demonstrate that uniform poly(vinyl butyral) (PVB) fibres can be safely electrospun onto a monolayer of human dermal fibroblasts using a portable device.

**Results:**

PVB in solvent mixtures containing various amounts of ethanol and water was electrospun. Six percent (weight-to-volume ratio) PVB in a 9:1 ethanol:water ratio was the solution with the highest content in water that could be electrospun into consistent fibres with an average diameter of 0.9 μm (± 0.1 μm). Four and five percent PVB solutions created beaded fibres. A 8:2 ethanol:water solution lead to microbead formation while a 7:3 ethanol:water mix failed to fully dissolve. The selected solution was successfully electrospun onto a monolayer of human dermal fibroblasts and the process had no significant effect (*p* < 0.05) on cell viability compared to the control without fibres.

**Conclusions:**

PVB–ethanol–water solutions could be electrospun without damaging the exposed cell layer. However, further work is required to demonstrate the long-term effect of PVB as a wound healing material.

## Introduction

Wound healing materials have been studied extensively to aid the healing of both acute and chronic wounds. Acute wounds are caused by trauma and although these typically heal between 6 and 12 weeks (Zahedi et al. 2010) they are still a widespread problem with around 11 million people affected in the US alone (Singer and Dagum 2008; Demidova-Rice et al. 2013). On the other hand, chronic wounds show impaired healing due to infections. These include venous leg ulcers, diabetic foot ulcers and pressure ulcers (Moore et al. 2006). In the UK, around £1bn a year is spent by the National Health Service in wound management (Harding et al. 2002). This creates a large demand for wound healing materials.

Recently, electrospun fibres have been proposed as an alternative to the traditional gausses, films and gels used in wound healing. Briefly, electrospinning involves applying an electric field to a polymer solution, creating a charged jet that elongates and solidifies into fibres following solvent evaporation (Huang et al. 2003). Electrospinning can produce submicron fibres, which are much smaller compared to those created with ink jet printing, a more developed jet-based technology (Jayasinghe 2013). Using electrospun fibres for biomedical purposes could lead to several advantages. Firstly, the small holes and high surface area of the fibrous network promote the hemostasis phase of the wound healing process without the use of any hemostatic agent. Secondly, the small pore size of the mesh may both enable gaseous exchanges (for the cells) and prevent bacterial invasion. Furthermore, the high surface-to-volume ratio means that they could serve as a carrier for releasing bioactive molecules or cells. These can easily be incorporated into the fibre mesh through a co-axial spinning process (Zahedi et al. 2010) or by blending the molecules with the electrospun solution (Zeng et al. 2003). Finally, electrospun materials also have the ability to provide physical cues to local cells, by mimicking the extracellular matrix (Sell et al. 2007).

As wound healing materials, electrospun mesh could be used off-the-shelf (being spun beforehand), or they could be spun directly onto the wound surface. The former enables better control over the fibre diameter and density. However, this approach becomes particularly challenging when working with thin fibre meshes, as damage can occur during handling. Moreover, if bioactive molecules are incorporated, post-processing steps and storage may cause these biomolecules to degrade or release prematurely. In situ spinning, i.e. directly onto the wound site, may overcome these limitations. Moreover, it may facilitate conformability to the wound site and easily fit into 3D contours (Zahedi et al. 2010).

Electrospinning setups normally require a large working space in order to accommodate most of the components. These include a high-voltage device, a syringe pump and a set of electrodes. The setups are very cumbersome and direct spinning onto a wound site might prove challenging, in particular in remote locations. Portable, light and battery-powered devices could ease this process. Although currently such devices are not available commercially they have been developed in several research studies. Xu et al. (2015) developed a battery-powered handheld device that is finger-pressed to spin medical glue fibres. The fibres produced were shown to stop bleeding on a wound on a rat. In another study, solar cell and a hand power generator were incorporated into a portable device in order to improve flexibility (Yan et al. 2015). Both of the aforementioned devices do not have an in built syringe pump and require to be finger-pressed which could have variable flow rates. More recently, Haik et al. (2017) used a handheld device, developed by Nicast Ltd. (Israel), to directly spin 4 different nanofiber dressings onto superficial partial thickness wounds with a flow rate of 4.5 ml/h. The clinician was able to fully cover the wound by moving the device in uniform motion during spinning. However, the use of both hands was required to hold the device, while aiming electrospun fibres towards the wound area. In a previous study, our research group has developed a small portable battery-operated electrospinning device that uses a linear actuator to precisely control delivery from cartridges (Mouthuy et al. 2014). The system also includes an optional pen extension that can increase the flexibility of the angle and direction of spinning. The small size and light weight of the pen allowed it to be aimed at the wound surface with the use of only one hand. The device was successfully used to electrospin common polymers used in electrospinning such as poly(vinyl butyral) (PVB), poly(*p*-dioxanone), polycaprolactone and poly(ethylene oxide). We also demonstrated direct spinning onto skin and shown that the quality of the fibres produced with the device were comparable to those created with a benchtop machine.

Among the polymers spun with our device, PVB caught our attention because of its ability to dissolve in ethanol but not in water (Yener and Jirsak 2012). Moreover, PVB has been shown to possess biocompatibility and non-toxicity (Posavec et al. 2011). We previously used an ethanol–methanol mixture to dissolve the polymer (Mouthuy et al. 2014). However, the use of methanol is potentially toxic and an irritant to the patient and the person applying the mesh (if different). This toxicity can be caused either by inhalation of solvent vapours or through contact of the cells with residual solvent in the fibres (Bock et al. 2012).

The aim of this study was to investigate whether PVB fibres could be reliably and safely electrospun onto a cell monolayer in vitro, with our portable electrospinning device. To achieve this, our first objective was to prepare various solutions of PVB dissolved in mixtures of ethanol and water, and to identify a candidate for in situ electrospinning. Our second objective was then to demonstrate that the electrospun fibres spun with our selected PVB solution had no adverse effect on the viability of primary human dermal fibroblasts (HDFs) monolayer.

## Materials and methods

### Preparation of polymer solutions

Poly(vinyl butyral) (PVB, also referred to as Mowital) B75H was kindly donated by Kuraray Europe (Germany). PVB was dissolved at a concentration between 4 and 6% (weight to volume ratio) in an ethanol–water mix. The ethanol:water ratios investigated included 10:0, 9.5:0.5, 9:1, 8:2 and 7:3. All solutions were agitated at room temperature on a roller for at least 24 h prior to electrospinning.

### Electrospinning on static plates

Electrospinning was carried out using a handheld electrospinning device developed and described in a previous publication (Mouthuy et al. 2014). Briefly, the device contains a linear actuator to form a syringe pump and a converter model to provide high voltage. A pack of 10 rechargeable AA batteries powered the device. A pen extension could be attached onto the positive electrode through a Teflon tube. A metal plate was used as the ground electrode.

### Scanning electron microscopy (SEM)

The fibres were imaged using SEM as described in our previous study. Samples were mounted on an aluminium stub using a carbon adhesive disk and then gold coated using a SC7620 Mini Sputter Coater System (Quorum Technologies, Ltd, UK). High-resolution images were taken using an environment SEM (Carl Zeiss Evo LS15 Variable Pressure, Germany). Six areas of the fibre mesh were observed and fibre diameters (5 per area, 30 measurements in total) were measured using ImageJ (National Institutes of Health, US) software.

### Mesh area

A picture of the fibre mesh was taken beside a ruler acting as a scale. Samples were spun in triplicates and the mesh area was estimated through ImageJ using the freehand selection tool.

### Cell culture

Primary adult Human Dermal Fibroblasts (HDFs, Catalog number C0135C) were purchased from ThermoFisher Scientific (USA). The cells were grown on 14.5-cm-diameter petri dishes with Dulbecco Modified Eagle’s Medium, 10% foetal bovine serum and 1% Penicillin/Streptomycin. The medium was exchanged every 3 days. Cells were used for the experiments at 80% confluency.

### Electrospinning onto cell monolayers

To perform electrospinning on the cell monolayer, the culture medium was removed from the dish and 1 ml of fresh medium was added to prevent cells from dehydrating. The selected polymer solution (6% PVB B75H, in 90% Ethanol) was electrospun onto the moist cell layer for 1 min using the pen extension with a voltage of 13 kV with a flow rate of 0.8 ml/hr and an electrode distance of 20 cm. A wire placed in contact with the cell monolayer was used as the ground electrode. The fibres were observed before rehydrating the cells with media once more. A control specimen of cells where electrospinning was not conducted also had its media removed and 1 ml of fresh media added for 1 min. The setup was shown in Fig. 2a.

### Trypan blue assay

After the incubation time in the cells, the fibres were removed from the cells together with the media. Cells were detached via Trypsin/EDTA and centrifuged. Trypan blue from Invitrogen (USA) was added to an aliquot of the cell suspension in a 1:1 volume ratio and the cell viability was measured.

### Live/dead assay

Live/dead assay kit was purchased from Life Technologies (USA). The staining was performed 30 min after electrospinning according to the manufacturer’s instructions. Briefly, 4uM of EthD-1 solution and 2uM of Calcein AM solution were added to PBS. The solution was added to the cell monolayer and incubated for 30 min at room temperature away from light. The cells were imaged using a Nikon TiE 2000 fluorescence microscope (Japan). Calcein AM stained live cells green while dead cells were stained red with EthD-1.

### Statistical analysis

A one-way Anova (*p* < 0.05) was used to analyse statistical significance between fibres created from different parameters.

Two-way Anova (*p* < 0.05) was used to determine difference in HDF cell viability following electrospinning on the cell monolayers.

## Results

### Electrospinability of PVB Solutions

**Fig. 1:**
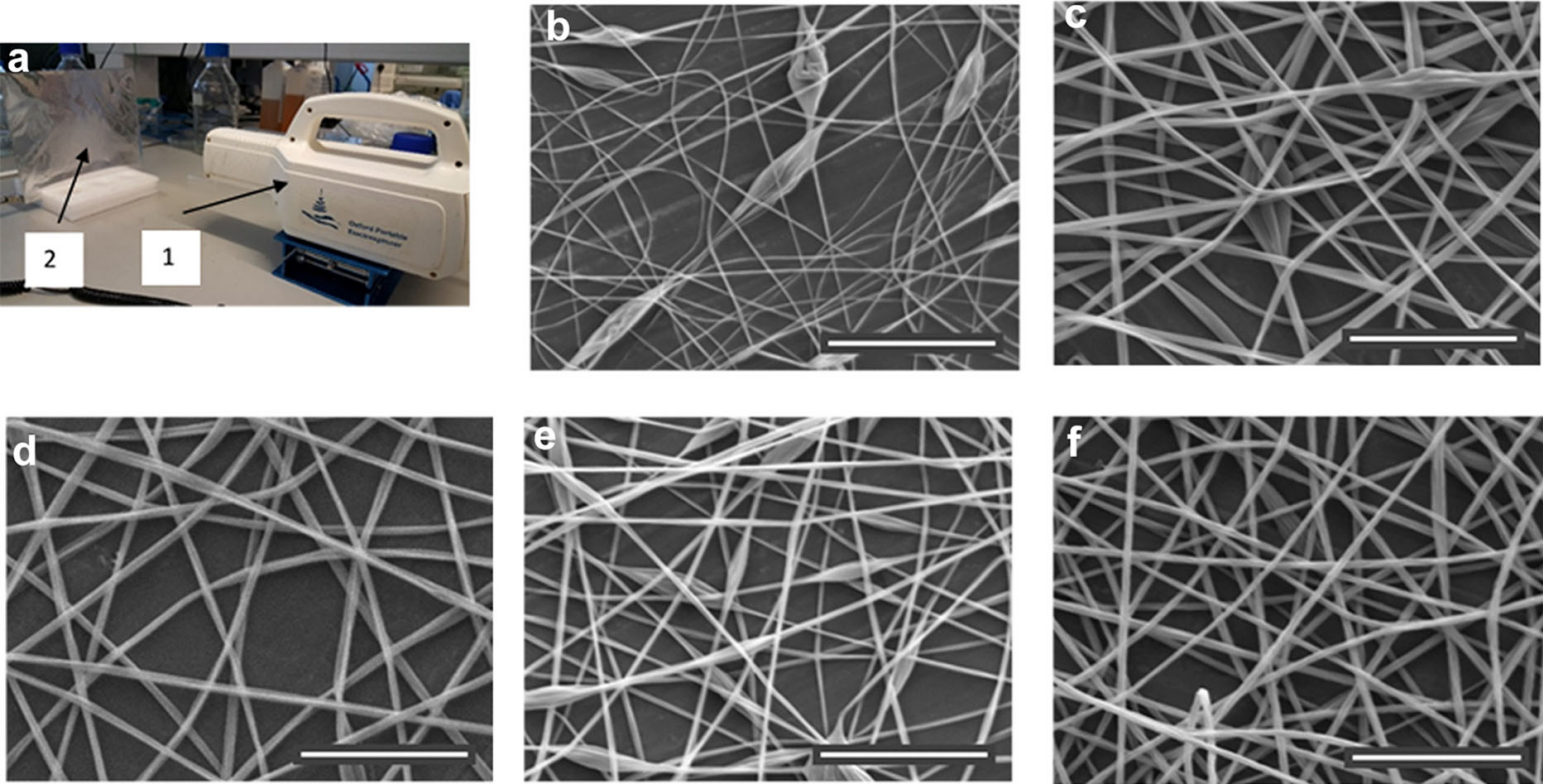
PVB fibres electrospun with the portable device on a metal plate from solutions with different PVB concentrations and with different ratios of ethanol and water (ethanol:water). **a** Electrospinning setup showing the portable device (1) and the metal plate used as the collector (2); **b** Fibres spun with 4% PVB in a 10:0 mix; **c** Fibres spun with 5% PVB in a 10:0 mix; **d** Fibres spun with 6% PVB in a 10:0 mix; **e** Fibres spun with 6% PVB in a 9.5:0.5 mix; **f** Fibres spun with 6% PVB in a 9:1 mix. Beads are observed in **b**, **c**, **e**. Scale bars show 20 µm

The results of the electrospinning experiments performed with the PVB solutions are presented in Table 1 and Fig. 1.

**Table 1:** Electrospinning parameters for PVB fibres

Voltage (kV)	Flow rate (ml/hour)	Electrode distance (cm)	Spinning time (s)	PVB in solvent (% w/v)	Ethanol:water ratio (v/v)	Fibre diameter ± SD (µm)	Fibre area ± SD (cm^2^)
13	0.8	20	60	4	10:0	0.4 ± 0.1	42 ± 3
13	0.8	20	60	5	10:0	1.1 ± 0.2	33 ± 1
13	0.8	20	60	6	10:0	1.2 ± 0.2	23 ± 2
13	0.8	20	60	6	9.5:0.5	0.7 ± 0.2	28 ± 2
13	0.8	20	60	6	9:1	0.9 ± 0.1	35 ± 1
13	0.8	20	60	6	8:2	N/A	N/A
13	0.8	20	60	6	7:3	N/A	N/A

*N/A* not applicable, electrospinning was not possible

Although fibres were created at 4, 5 and 6% of PVB in a 10:0 ethanol solution (shown in Fig. 1b, c, d, respectively) beaded fibres were observed at the two lower concentrations. Carrying on with the 6% PVB solutions, we showed that, fibres could be created with up to 10% water (solvent mix 9:1). With more water, electrospraying occurred (8:2) or the PVB failed to dissolve (7:3). Fibre diameter decreased significantly from 1.2 µm to 0.7 µm as the water content increased from a solvent mix of 10:0 to 9.5:0.5; however, the fibres created in the latter were beaded (Fig. 1e). As the water content decreased even further (solvent mix 9:1), the fibre diameter increased significantly back up to 0.9 µm and unbeaded fibres were formed (Fig. 1f). It is interesting to note that the fibre deposition area decreased with increasing PVB concentration and increased with the water content (Table 1).

The voltage selected for these experiments was 13 kV for all solutions. This corresponds to the highest voltage that the device can reach due to the choice of HV converter and to the battery capacity. The use of lower voltages did not lead to fibre formation and only led to the formation of large droplets.

### Electrospun fibres on human dermal fibroblasts

**Fig. 2:**
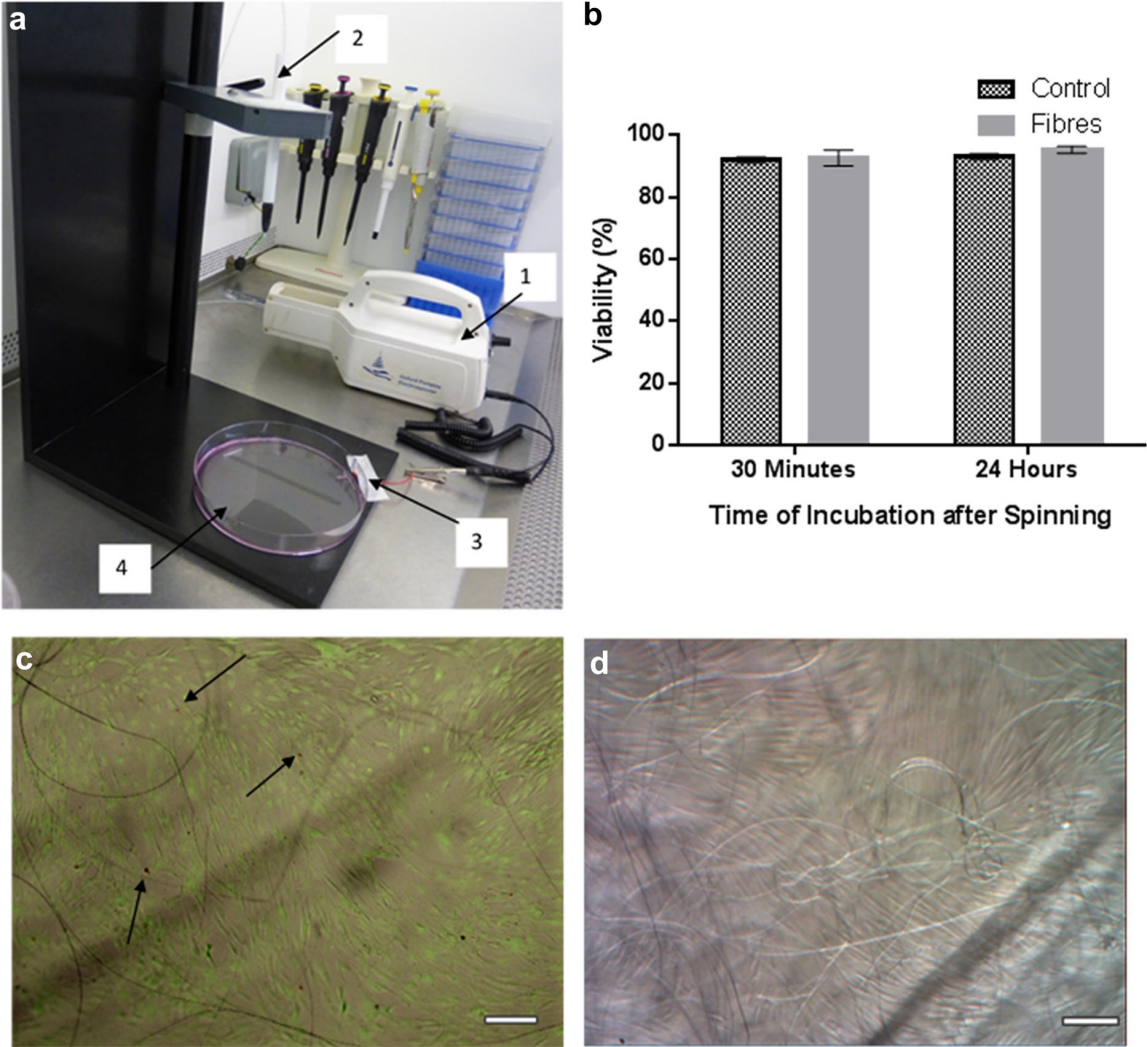
Electrospinning of a solution of 6% PVB in a 9:1 ethanol:water mix onto a mononolayer of human dermal fibroblasts (HDFs) using a portable device. **a** Setup of the experiment showing the portable device (1), the pen extension (2), the ground wire immersed in culture media (3) and the Petri dish with the HDFs monolayer (4); **b** Cell viability via Trypan blue observed after 30 min and 24 h following electrospinning; **c** Live/dead stain of HFD cells with fibres 30 min after spinning (green: live cells, red: dead cells, dead cells are shown by the arrows); **d** PVB fibres seen over a monolayer of HFDs 24 h after spinning under an optical microscope. Scale bars show 200 μm

Six percent PVB in a 9:1 mix were electrospun onto human dermal fibroblasts. The results are indicated in Fig. 2. Electrospinning did not influence significantly the viability of the cells compared to the control group (no electrospinning) for both 30 min and 24 h after spinning (Fig. 2b). Moreover, most of the cells were seen to be green fluorescent indicating live cells (Fig. 2c). The appearance of the dead cells (red stain) seemed to be rare and random.

## Discussion

Using solutions that are safe for both the patient and the device user is an important consideration for future uses of portable electrospinning devices in wound healing applications. Several hydrophilic polymers such as PEO and PVA can easily be electrospun into fibres from water-based solutions (Ding et al. 2010; Mouthuy et al. 2014). However, because they easily dissolve in water, these are not suitable for wound healing applications. The moisture on the wound would quickly disintegrate the fibres, making them useless as a healing material. PVB on the other hand, which results from the condensation of PVA with butyraldehyde, is hydrophobic, does not dissolve in water and can make use of ethanol to be put in solution (Xu et al. 2013). We have previously shown that PVB can be electrospun with our portable device using ethanol and methanol as solvents (Mouthuy et al. 2014). Here, we have successfully electrospun PVB fibres with the same device, using of a mixture of ethanol and water as the solvents. This is of significant importance as it avoids the use of toxic methanol.

In our experiments, we observed a significant increase in the fibre diameter as the concentration of PVB was increased from 4% (0.4 µm) to 6% (1.2 µm). This is a very typical observation (Li and Wang 2013). Moreover, beaded structures were observed at lower concentrations. This beading behaviour is also typically observed and is indicative of a transition towards the electrospraying mode, when the viscosity of the solution becomes too low to enable the jet to elongate into fibres (Fong et al. 1999). Beaded structures affect the overall porosity and are often seen as a defect in electrospun meshes.

Interestingly, adding water to the PVB solution resulted in a decrease of the fibre diameter. This is in opposition to a study by Yener et al., who showed that the fibre diameter increases with the water content due to an increase in viscosity, surface tension and conductivity (Yener and Jirsak 2012). A possible explanation for the difference in observations is the difference in the electrospinning setup. In particular, in the study by Yener et al., a voltage as high as 80 kV was used, while the distance was the same (20 cm). This means that much higher electric potentials were used compared to our study. In electrospinning, the effect of electric potential on the fibre diameter is variable (Li and Wang 2013). An increase in electric potential allows for more elongation of the fibres due to higher mutual repulsive forces hence decreasing the fibre diameter. On the other hand, mutual repulsive forces could also push more solution through hence creating larger fibres.

At 6% PVB, increasing the water content from 10:0 to 9.5:0.5 caused fibres to become beaded. However, a further increase to 9:1 led back to uniform fibres. This could be due to the increased conductivity caused by the water content allowing the spinning to slowly transition to electrospraying forming beaded fibres (Yener and Yalcinkaya 2013). However, as the water content increases, the increase in viscosity outweighs the conductivity and hence the solution was able to stretch out into fibres once more. Increasing the content of water in the ethanol solution was desirable to decrease the potential deleterious drying effect that ethanol could have at the wound site. Increasing the water content to 7:3 ethanol:water ratio, resulted in the PVB failing to dissolve and forming a gel-like structure within the solution possibly due to the hydrophobic PVB precipitating in the presence of water molecules.

As our target was to achieve a consistent fibrous mesh without beads and the highest content in water, the solution selected for the cell work was the 6% PVB in a 9:1 ethanol:water mix.

In our study, the chosen solution was successfully electrospun into fibres on the HDF monolayer in vitro. Moreover, we have observed that the cell viability was not affected by the deposition of fibres. The short-term viability test (30 min after spinning) showed that there were no necrotic effects caused by the electrospinning process such as stresses due to the high voltage or the impact of fibre deposition. This was expected since reports about cell electrospinning experiments (where cells are spun together with the polymer to avoid the need for manual seeding) have shown that the high voltages do not damage cells during the spinning (Townsend-Nicholson and Jayasinghe 2006; Jayasinghe 2013). This is thought to be because electrospinning uses a relatively low current which does not cause electroporation in cells. The longer term viability test (24 h after spinning) indicated that the electrospinning process caused no adverse effects on cell viability that could be less immediate, such as the toxic effect from residual ethanol that could lead to apoptosis. It is worth noticing that only a small number of fibres can be observed in Fig. 2c, d. This is due to the fact that the fibres lifted up upon re-addition of media, and some fibres were removed from the focus of the microscope’s objective.

We do not anticipate that ethanol-based solutions would become a safety issue for medical uses. A review study (Lachenmeier’s (2008)) concluded that ethanol absorption through the wounds via pharmaceutical preparations is not toxic to adults. Furthermore, it was found in another study that a high-frequency use and exposure to alcohol-based hand sanitizers or surgical scrubs for hand and skin hygiene do not cause significant risks of toxicity (Maier et al. 2015). In addition, amounts of residual solvent in electrospun meshes would be lower than in most ethanol-based products in both of the studies described above. Therefore, we believe that residual ethanol from the fibre mesh would not cause toxicity. In our solutions, we have also managed to incorporate small amounts of water. This may help to limit the risk of drying of the wound with the residual ethanol (The National Institute for Occupational Safety and Health 2014).

This study has several limitations. First of all, the maximum voltage that could be applied during spinning was l3 kV. This is due to the physical limits of the converter used in the portable device. Investigating higher voltages may have prevented the formation of beads in some of the polymer solutions. Additionally, this could have allowed us to achieve smaller fibre diameters. Secondly, the spinning duration was fixed to 1 min. Although this was long enough to deposit a relatively dense network of fibres on the collector, the pores sizes are still relatively large, being above the micron (see SEM images in Fig. 1). These gaps are large enough for bacteria to enter and hence such fibre density would not be suitable for such a role. A wider range of spinning duration will be investigated in the future. Thirdly, we have not investigated the long-term effect of PVB fibres on cells. Although PVB nanobeads had been shown to lack cytotoxicity in vitro (Posavec et al. 2011), little is known about how cells behave on PVB meshes. Examining the cell interaction with the fibres including testing the viability, proliferation, differentiation and extracellular matrix secretion of cells will help to elaborate on its potential in wound healing. Finally, it is worth mentioning that spinning was performed in a Petri dish, which poorly represents a wound, both physically and biologically. Future work should also explore the use of the device and the solution to treat defects created in an in vivo model.

## Conclusions

We have successfully electrospun, PVB fibres using ethanol:water mixtures using a portable device. The absence of methanol, which is used in most previous studies, reduces the potential cell toxicity of the solution. Our results demonstrated that a solution containing a 9:1 ethanol:water ratio could be electrospun onto HDFs monolayers without affecting the cell viability. Although further work needs to be performed on cell-interactions and long-term culture with the fibres, these results suggest that viable, uniform PVB–ethanol–water fibres could be spun directly onto a wound without damaging the exposed cell layer. This supports further the potential of portable electrospinning devices for medical applications.

## References

[CR1] Bock N, Dargaville TR, Woodruff MA (2012). Electrospraying of polymers with therapeutic molecules: State of the art. Prog Polym Sci.

[CR2] Demidova-Rice Tatiana N, Hamblin MR, Herman IM (2013). Acute and impaired wound healing: pathophysiology and current methods for drug delivery, part 1: normal and chronic wounds: biology, causes, and approaches to care. Adv Skin Wound Care.

[CR3] Ding W, Wei S, Zhu J, Chen X, Rutman D, Guo Z (2010). Manipulated electrospun PVA nanofibers with inexpensive salts. Macromol Mater Eng.

[CR4] Fong H, Chun I, Reneker DH (1999). Beaded nanofibers formed during electrospinning. Polymer.

[CR5] Haik J, Kornhaber R, Blal B, Harats M (2017). The feasibility of a handheld electrospinning device for the application of nanofibrous wound dressings. Adv Wound Care.

[CR6] Harding KG, Morris HL, Patel GK (2002). Clinical review Healing chronic wounds. Br Med J.

[CR7] Huang ZM, Zhang YZ, Kotaki M, Ramakrishna S (2003). A review on polymer nanofibers by electrospinning and their applications in nanocomposites. Compos Sci Technol.

[CR8] Jayasinghe SN (2013). Cell electrospinning: a novel tool for functionalising fibres, scaffolds and membranes with living cells and other advanced materials for regenerative biology and medicine. Analyst.

[CR9] Lachenmeier DW (2008). Safety evaluation of topical applications of ethanol on the skin and inside the oral cavity. J Occup Med Toxicol.

[CR10] Li Z, Wang C (2013). Effects of working parameters on electrospinning. One-dimensional nanostructures.

[CR11] Maier A, Ovesen JL, Allen CL, York RG, Gadagbui BK, Kirman CR, Poet T, Quinones-Rivera A (2015). Safety assessment for ethanol-based topical antiseptic use by health care workers: Evaluation of developmental toxicity potential. Regul Toxicol Pharmacol.

[CR12] Moore K, McCallion R, Searle RJ, Stacey MC, Harding KG (2006). Prediction and monitoring the therapeutic response of chronic dermal wounds. Int Wound J.

[CR13] Mouthuy P, Groszkowski L, Ye H (2014). Performances of a portable electrospinning apparatus. Biotechnol Lett.

[CR14] National Institute for Occupational Safety and Health (2014) CDC—ETHANOL (ANHYDROUS)—International Chemical Safety Cards. https://www.cdc.gov/niosh/ipcsneng/neng0044.html. Accessed 6 Nov 2017

[CR15] Posavec D, Dorsch A, Bogner U, Bernhardt G, Nagl S (2011). Polyvinyl butyral nanobeads: preparation, characterization, biocompatibility and cancer cell uptake. Microchim Acta.

[CR16] Sell S, Barnes C, Smith M, McClure M, Madurantakam P, Grant J, MacManus M, Bowlin G (2007). Extracellular matrix regenerated: tissue engineering via electrospun biomimetic nanofibers. Polym Int.

[CR17] Singer AJ, Dagum AB (2008). Current management of acute cutaneous wounds. N Engl J Med.

[CR18] Townsend-Nicholson A, Jayasinghe SN (2006). Cell electrospinning: a unique biotechnique for encapsulating living organisms for generating active biological microthreads/scaffolds. Biomacromol.

[CR19] Xu H, Li H, Chang J (2013). Controlled drug release from a polymer matrix by patterned electrospun nanofibers with controllable hydrophobicity. J Mater Chem B.

[CR20] Xu S-C, Qin C-C, Yu M, Dong R-H, Yan X, Zhao H, Han W-P, Zhang H-D, Long Y-Z (2015). A battery-operated portable handheld electrospinning apparatus. Nanoscale.

[CR21] Yan X, Yu M, Zhang L-H, Jia X-S, Li J-T, Duan X-P, Qin C-C, Dong R-H, Long Y-Z (2015). A portable electrospinning apparatus based on a small solar cell and a hand generator: design, performance and application. Nanoscale.

[CR22] Yener F, Jirsak O (2012) Effect of nonsolvent on electrospinning performance and nanofibre properties. In Conference proceedings book 4th international conference nanocon 2012, Brno, 23–25 Oct 2012, pp 471–475

[CR23] Yener F, Yalcinkaya B (2013). Electrospinning of polyvinyl butyral in different solvents. E-Polymers.

[CR24] Zahedi P, Rezaeian I, Ranaei-Siadat SO, Jafari SH, Supaphol P (2010). A review on wound dressings with an emphasis on electrospun nanofibrous polymeric bandages. Polym Adv Technol.

[CR25] Zeng J, Xu X, Chen X, Liang Q, Bian X, Yang L, Jing X (2003). Biodegradable electrospun fibers for drug delivery. J Control Release.

